# Effects of 12 Weeks of Daily Melatonin Administration on Inflammatory Markers and Adipose Tissue Mass of Rats under Hypoestrogenism

**DOI:** 10.3390/medicina60050740

**Published:** 2024-04-29

**Authors:** Taciane Maria Melges Pejon, Guilherme Borges Pereira, Cynthia Aparecida de Castro, Fernanda de Freitas Anibal, Wladimir Rafael Beck

**Affiliations:** 1 Laboratory of Endocrine Physiology and Physical Exercise, Department of Physiological Sciences, Federal University of São Carlos, São Carlos 13565-905, Brazil; tacianepejon92@gmail.com; 2 Laboratory of Clinical Exercise Physiology, Department of Physiological Sciences, Federal University of São Carlos, São Carlos 13565-905, Brazil; gbp.ufscar@gmail.com; 3 Laboratory of Pathology and Biocompatibility, Department of Morphology and Pathology, Federal University of São Carlos, São Carlos 13565-905, Brazil; cynthia.castro@ufscar.br; 4 Laboratory of Inflammation and Infectious Diseases, Department of Morphology and Pathology, Federal University of São Carlos, São Carlos 13565-905, Brazil; ffanibal@ufscar.br

**Keywords:** estrogen deficit, N-acetyl-5-methoxytryptamine, inflammation, white adipose tissue, interleukins, cytokines

## Abstract

*Background and Objectives*: The hormonal state of hypoestrogenism is associated with the accumulation of white adipose tissue, which can induce an increase in pro-inflammatory markers, leading to progressive health complications. Melatonin can act on adipose tissue mass, promoting its reduction and influencing inflammation, reducing IL-6 and releasing IL-10, pro- and anti-inflammatory markers, respectively. However, the role of melatonin regarding such parameters under the context of hypoestrogenism remains unknown. The aim of this study was to determine the effect of 12 weeks of hypoestrogenism and melatonin on white adipose tissue mass and circulating levels of IL-6, IL-10, TGF-β-1, and leukotriene C4 (LTC4). *Materials and Methods*: The animals (Wistar rats with sixteen weeks of age at the beginning of the experiment) under hypoestrogenism were submitted to the surgical technique of bilateral ovariectomy. The animals received melatonin (10 mg·kg^−1^) or vehicles by orogastric gavage every day for 12 weeks and administration occurred systematically 1 h after the beginning of the dark period. White adipose tissue (perigonadal, peritoneal, and subcutaneous) was collected for mass recording, while blood was collected for the serum determination of IL-6, IL-10, TGF-β-1, and LTC4. *Results*: Hypoestrogenism increased the perigonadal and subcutaneous mass and IL-6 levels. Melatonin kept hypoestrogenic animals in physiological conditions similar to the control group and increased thymus tissue mass. *Conclusions*: Hypoestrogenism appears to have a negative impact on white adipose tissue mass and IL-6 and although melatonin commonly exerts a significant effect in preventing these changes, this study did not have a sufficiently negative impact caused by hypoestrogenism for melatonin to promote certain benefits.

## 1. Introduction

The significant decrease in estrogen levels inevitably occurs in the menopausal phase of woman or by surgical intervention to remove the ovaries. This hormonal-deficit condition progressively leads to health impairments due to numerous physiological changes previously regulated by estrogens [1]. Among these changes, which commonly involve energetic metabolic imbalances [2,3], the accumulation of white adipose tissue can be highlighted, being linked to the regulatory mechanisms through the estrogen receptors present in this tissue [4,5]. Although the function of adipose tissue in energy storage and generation is well known, the excess predisposes to obesity and other metabolic diseases over time; it can also induce inflammation [6]. 

This inflammation is characterized by the release of interleukins and cytokines by adipocytes, such as interleukin-6 (IL-6) and interleukin-10 (IL-10), in addition to the transforming growth factor (TGF-β1) [6]. The TGF-β family is related to the reduction in adipogenesis, and may have its levels increased with excessive fat accumulation [7]. IL-6 is considered pro-inflammatory and increased levels in the bloodstream can culminate in metabolic diseases [8]. Acute and chronic inflammation responses can also cause serum leukotriene elevations, increasing the risk of atherosclerosis [9]. In contrast, IL-10 is released by adipocytes in an attempt to neutralize inflammation in a compensation mechanism, acting as an anti-inflammatory factor [10]. Although the propensity of the hypoestrogenism state to induce the accumulation of white fat is extremely recognized, the influence on these markers is not conclusive when considering reports [6,11], requiring further investigations to understand if there is a relationship between these parameters.

Thus, it is important to seek possible strategies to control complications that may occur in a state of hypoestrogenism. Hormone replacement therapy is commonly applied, but the health risks associated with hormone replacement of these steroids invite questions of its use; the risk of breast cancer is one of the most alarming, which encourages the search for safer alternatives [12]. Melatonin, a hormone synthesized by the pineal gland, has a broad physiological action, participating positively in bone [13,14] and muscle maintenance [15], among other target tissues. Like sex hormones, their secretion gradually reduces over the years and impacts their physiological functions. So, the exogenous use of melatonin has been increasing in numerous areas for its health benefits, mainly due to the lack of reports on its serious adverse effects, being characterized as safe even in high doses [16,17], including in studies involving hypoestrogenism [17,18]. In addition, there seems to be a presentation of positive expectations due to its role in energy metabolism [19] and its participation in inflammatory processes [20]. With regard to white adipose tissue, studies have demonstrated the ability of melatonin to regulate lipolysis and suppress adipogenesis, influencing fat deposition and consequently reducing tissue mass [21]. As for the context of inflammation, melatonin may reduce pro-inflammatory mediators like IL-6 [8,22], while it can interfere with the production of leukotrienes from the reduction of 5-lipoxygenase [23] and stimulates the release of anti-inflammatory cytokines such as IL-10 in response to a challenge [20]. 

When reconciling the administration of melatonin with hypoestrogenism, there is controversial evidence about its action in reducing the total mass of adipose tissue [24,25], which may be dependent on the dosage and time of administration, making it relevant to continue studies on this topic. Also, evaluating pro- and anti-inflammatory markers in serum, beyond a more applicable clinical measure, could also contribute to understand the role of melatonin in the relationship between adipose tissue accumulation and the release of these markers. For this purpose, the aim of this study was to determine the impact of 12 weeks of hypoestrogenism in association with melatonin daily administration on white adipose tissue mass and circulating levels of IL-6, IL-10, TGF-β1, and LTC4.

## 2. Materials and Methods

### 2.1. Animals and Environmental Conditions

We evaluated forty female Wistar rats (body mass: 267 ± 27 g at the beginning of the experiment) obtained from the central Bioterium of the Federal University of São Carlos and subsequently housed in the Bioterium of the Laboratory of Endocrine Physiology and Physical Exercise. The Bioterium follows a light/dark cycle of 12/12 h and throughout the experiment, the environmental conditions were strictly controlled, including the temperature (22 ± 2 °C) and relative humidity (45–55%). The animals received specific pelleted feed for rodents (Presence^®^, Descalvado, SP, Brazil) and water ad libitum throughout the experiment. During the light cycle, Led lamps (2700 K; 565–590 nm; 60 lux) were used. For the dark phase, the animals remained in total darkness and the interventions that occurred during this period were performed in an environment with reflectors installed with a red filter (LEE Filters, #fire19; 600 nm; <15 lux) [26,27,28]. The experimental procedure was conducted in accordance with the Guide for the Care and Use of Laboratory Animals, and maximum care was ensured to minimize the number of animals used and their suffering in this study. The experiment was approved by the Ethics Committee on Animal Use (CEUA) of the Federal University of São Carlos under protocol no. 1694190521.

### 2.2. Experimental Design

**Figure 1:**
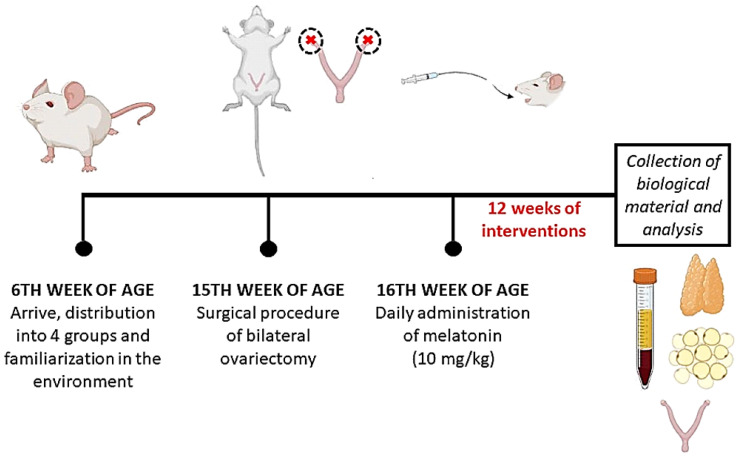
Experimental design of this study. mg: milligrams; kg: kilograms. Figure created in BioRender.com.

In the sixth week of age, the animals were randomly distributed into four groups: control + vehicle solution (CT: *n* = 10), ovariectomized + vehicle solution (OVX: *n* = 10), melatonin (MEL: *n* = 10), and ovariectomized with melatonin administration (OVX + MEL: *n* = 10). After familiarization with the environment, animals from the OVX and OVX + MEL groups were subjected to the surgical procedure of bilateral ovariectomy in the fifteenth week of age. In the sixteenth week of age of the animals, the melatonin administration protocol lasting 12 weeks was initiated (MEL and OVX + MEL) (Figure 1).

### 2.3. Surgical Procedure of Bilateral Ovariectomy

The technique was preceded with intraperitoneal anesthesia: ketamine (10 mg·kg^−1^) and xylazine (0.1 mg·kg^−1^) for the ovariectomized groups (OVX and OVX + MEL). According to Zarrow et al. [29], a 1.5 cm incision was made on both sides in the skin, lateral to the spine, between the last rib and the knee, allowing the ovaries to be exposed, tied, and removed from the pelvic cavity. The remainder of the tissue returned to the peritoneal cavity and all the layers were sutured with cotton thread (line 10). The animals received analgesic (14.2 mg/kg of sodium dipyrone, 1× time per day for 3 days) and were kept under observation in the post-operative period, and the recovery process lasted for 1 week.

### 2.4. Melatonin Administration

The method of melatonin administration (SIGMA-ALDRICH, Co., St. Louis, MO, USA) was by orogastric gavage (BD-12 stainless steel needle) [30]. Over the course of 12 weeks, each animal received daily a solution of 10 mg/kg previously dissolved in ethanol (<0.1%) and diluted in filtered water. The solution with melatonin and the vehicle solution (containing <0.1% ethanol) were prepared daily and kept stored at 4 °C until the moment of administration (1 h after the beginning of the dark period).

### 2.5. Obtention and Storage of Biological Material

After the 12-week intervention, euthanasia was performed by decapitation, a method that can be used according to the American Veterinary Medical Association [31]. The blood sample was collected from the region of the body at which the animal was decapitated. Immediately after collection, the blood rested for 15 min (4 °C) and then was centrifuged for 15 min at 3000 RPM for serum separation and subsequently stored at −80 °C to the time of interleukin (6 and 10), LTC4, and TGF-β1 analyses. 

Perigonadal, subcutaneous, and peritoneal adipose tissues; the thymus; and the uterus were collected to record the total mass.

### 2.6. Biochemical Analysis

Serum levels of cytokines were determined using ELISAs (Enzyme-Linked Immunosorbent Assays) to interleukin 6 (IL-6), interleukin 10 (IL-10), and transforming growth factor beta 1 (TGF-β1). The method followed the specifications of corresponding BD Biosciences Pharmingen^®^ (IL-6: Cat. No. 550319; IL-10: Cat. No. 555134; TNF-α: Cat. No. 558535; San Diego, CA, USA) and R&D Systems (TGF-β1: Cat. No. DB100C; Minneapolis, MN, USA) kits. LTC4 was determined using Enzyme Immunoassay Analysis—EIA kits (Cat. No. 501070, Cayman Chemical Co.—Ann Arbor, MI, USA). The concentrations were calculated from the concentration curve of the cytokine patterns and expressed in pg/mL.

### 2.7. Statistical Analysis

The results are presented as the mean ± standard deviation. Data were submitted to Shapiro–Wilk’s normality test, allowing parametric statistics usage. Data from interleukins; LTC4; TGF-β1; perigonadal, subcutaneous, and peritoneal adipose tissues; the thymus; and the uterus were subjected to a two-way factorial analysis of variance for the main effects of hypoestrogenism (hypoestrogenism, two levels: CT and MEL vs. OVX and OVX + MEL) and melatonin (melatonin, two levels: CT and OVX vs. MEL and OVX + MEL). When appropriate, we used the Tukey–Kramer post hoc test. A significance level of 5% was established for all analyses, and Statistica 7.0 (StatSoft, Inc., Tulsa, OK, USA) was used.

## 3. Results

### 3.1. IL-6, IL-10, LTC4, and TGF-β1

**Figure 2:**
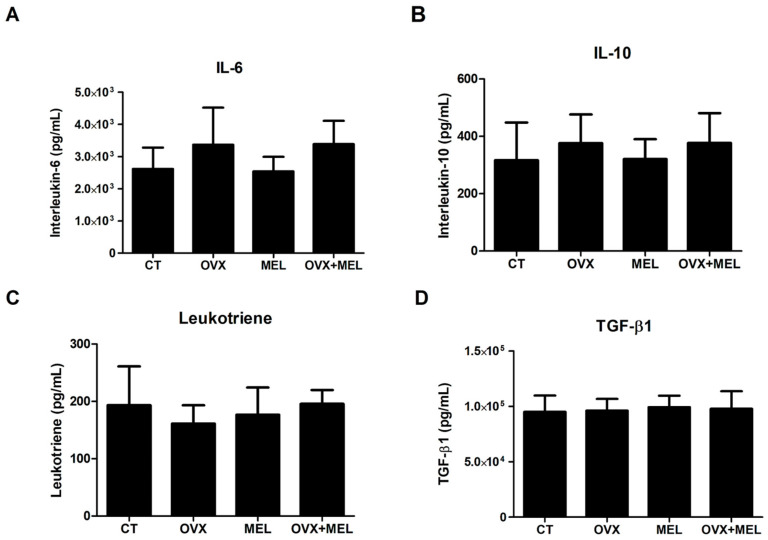
Data from IL-6 panel (**A**), IL-10 panel (**B**), LTC4 panel (**C**), and TGF-β1 panel (**D**) in control group (CT), ovariectomized group (OVX), melatonin group (MEL), and ovariectomized/melatonin group (OVX + MEL). Values expressed as mean and standard deviation. pg: picograms; mL: milliliters.

Hypoestrogenism increased IL-6 (F = 8.75; *p* < 0.01), while melatonin did not promote a difference (F = 0.009; *p* = 0.92). There was no difference for CT in relation to OVX + MEL (*p* = 0.12) (Figure 2A). IL-10 was not affected by hypoestrogenism and melatonin (F = 2.28; *p* = 0.14 and F = 0.004; *p* = 0.94, respectively) (Figure 2B).

There was no change in LTC4 for the effect of hypoestrogenism (F = 0.17; *p* = 0.67) or for melatonin (0.33; *p* = 0.56) (Figure 2C). In addition, hypoestrogenism and melatonin did not promote a difference in TGF-β1 (F = 0.001; *p* = 0.97 and F = 0.48; *p* = 0.49, respectively) (Figure 2D). 

### 3.2. White Adipose Tissue Mass

**Figure 3:**
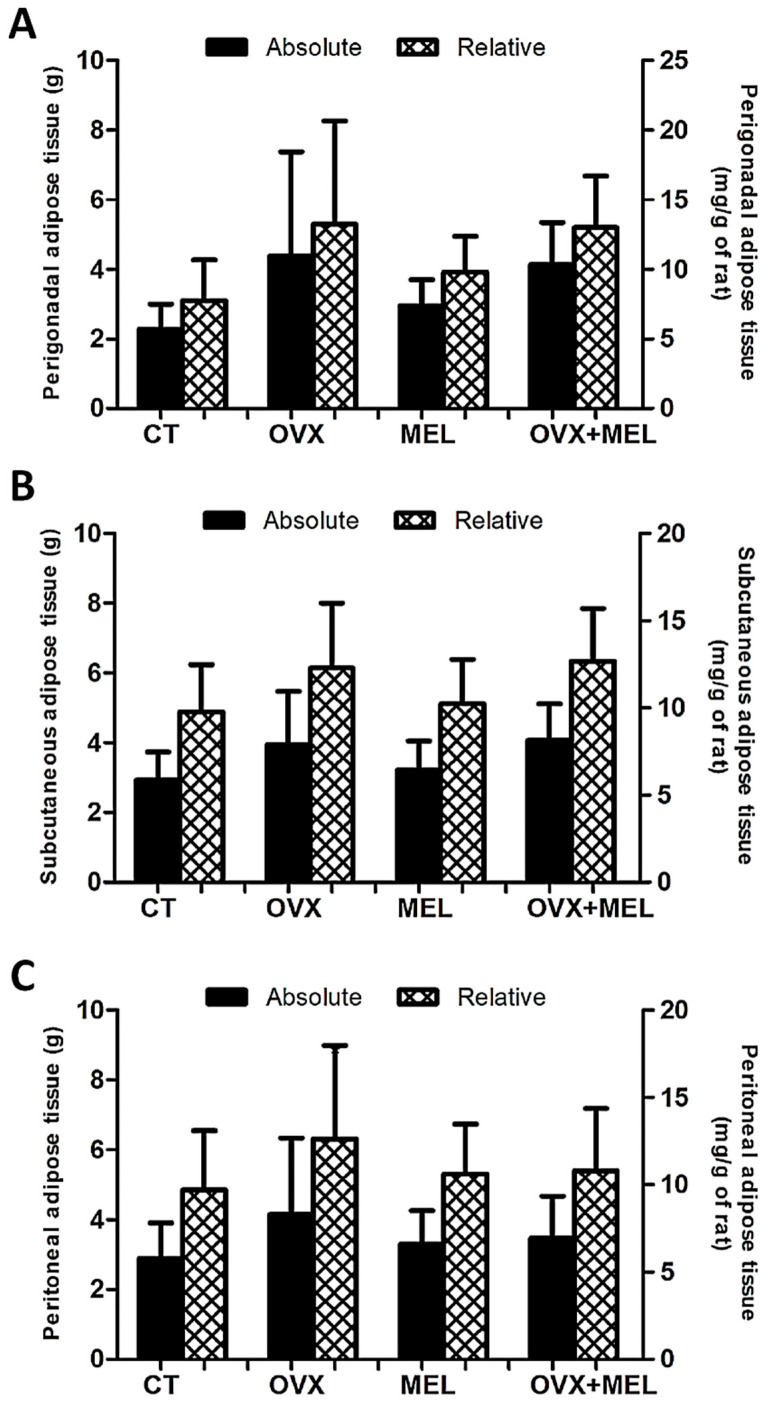
Data from absolute and relative tissue mass of the white adipose tissue in the control group (CT), ovariectomized group (OVX), melatonin group (MEL), and ovariectomized/melatonin group (OVX + MEL). Panel (**A**) represents the data of the perigonadal, panel (**B**) the data of the subcutaneous, and panel (**C**) the data of the peritoneal adipose tissue. Values expressed as the mean and standard deviation. g: grams; mg: milligrams.

Hypoestrogenism increased the perigonadal (F = 6.21; *p* < 0.05) and subcutaneous (F = 6.51; *p* < 0.05) adipose tissue mass, but did not cause changes in peritoneal fat (F = 1.97; *p* = 0.17). Melatonin did not cause significant differences in perigonadal (F = 0.09; *p* = 0.76), subcutaneous (F = 0.34; *p* = 0.56), and peritoneal (F = 0.06; *p* = 0.80) adipose tissue. In relation to relative mass of these parameters, hypoestrogenism promoted an increase in the perigonadal (F = 6.07; *p* < 0.05) and subcutaneous (F = 5.75; *p* < 0.05), although it did not present any difference in the peritoneal (F = 1.25; *p* = 0.27). Melatonin did not cause changes in perigonadal (F = 0.24; *p* = 0.62), subcutaneous (F = 0.17; *p* = 0.67), and peritoneal (F = 0.11; *p* = 0.74) relative mass (Figure 3).

### 3.3. Thymus Tissue Mass

**Figure 4:**
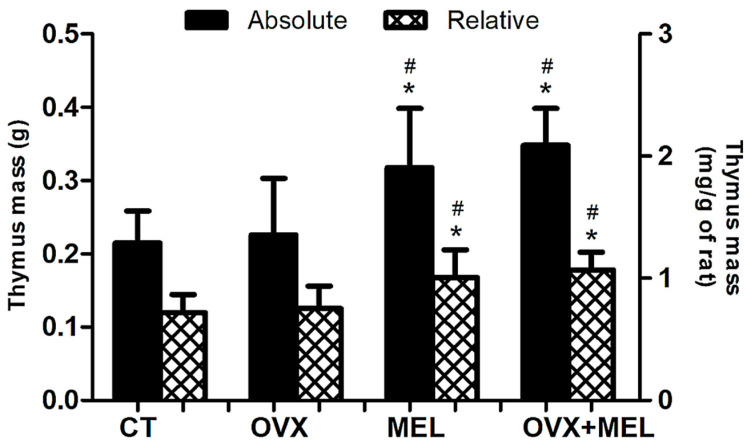
Data from absolute (Y-axis on the left) and relative (Y-axis on the right) tissue mass of the thymus in the control group (CT), ovariectomized group (OVX), melatonin group (MEL), and ovariectomized/melatonin group (OVX + MEL). Values expressed as the mean and standard deviation. The statistics provided in the graphs are the results of the Tukey–Kramer post hoc test: * *p* < 0.05 in relation to CT for the same variable; ^#^ *p* < 0.05 in relation to OVX for the same variable. g: grams; mg: milligrams.

The absolute mass of the thymus (F = 0.93; *p* = 0.34) and relativized by body mass (F = 0.64; *p* = 0.42) did not present an effect by hypoestrogenism, but melatonin promoted increasing in both measures (F = 27.30; *p* < 0.01 and F = 25.85; *p* < 0.01, respectively). The statistical differences between the groups are shown in Figure 4.

### 3.4. Uterus Mass

**Figure 5:**
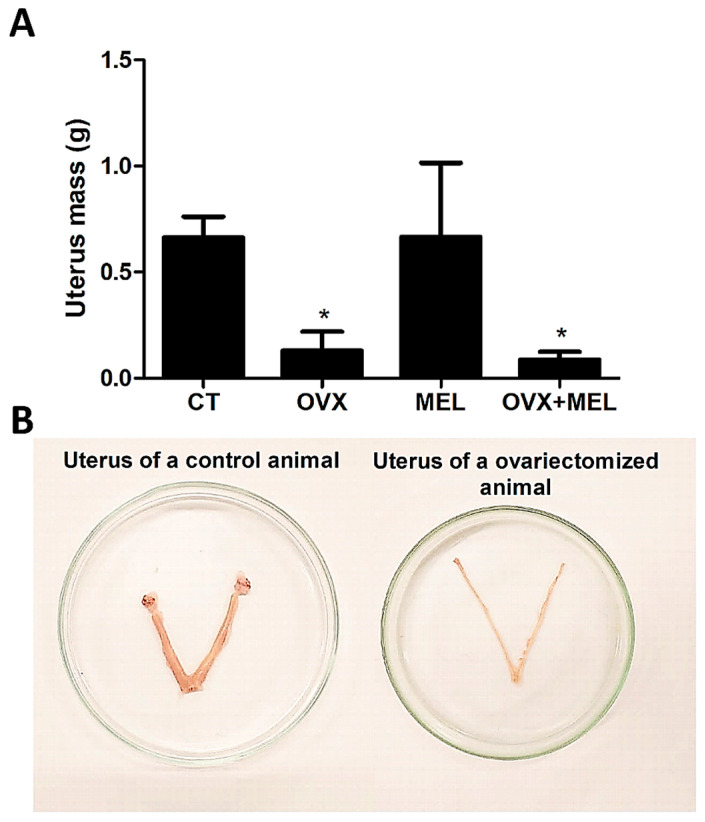
Data from absolute mass of the uterus panel (**A**) in the control group (CT), ovariectomized group (OVX), melatonin group (MEL), and ovariectomized/melatonin group (OVX + MEL). Values expressed as the mean and standard deviation. Panel (**B**) illustrates the difference between a uterus of a control animal (with presence of ovaries) and an animal submitted to the surgical technique of ovariectomy (ovaries removed). The statistics provided in the graphs are the results of the Tukey–Kramer post hoc test: * *p* < 0.05 in relation to CT for the same variable. g: grams.

Hypoestrogenism significantly reduced the absolute mass of the uterus (F = 78.48; *p* < 0.01), while melatonin did not cause changes (F = 0.10; *p* = 0.74) (Figure 5).

## 4. Discussion

The main findings of this study report that hypoestrogenism causes an increase in the mass of white adipose tissue, which may be related to the increase in IL-6, a pro-inflammatory marker. As a strategy to prevent alterations caused by hypoestrogenism, we daily administered melatonin for 12 weeks; melatonin has very few reports regarding eventual negative side effects and has some potential positive effects on such a scenario. However, no significant effects on these parameters were verified. Nevertheless, the OVX + MEL group did not present a statistical difference in relation to CT in several results, indicating that melatonin promoted physiological responses despite hypoestrogenism, preventing health complications that may be associated with the parameters evaluated in the course of time.

Initially, it is relevant to indicate the success of the ovariectomy surgery, which was confirmed from the uterine mass, demonstrating tissue atrophy at the end of the 12 weeks of interventions (*p* < 0.01 when comparing the OVX and OVX + MEL groups in relation to CT). With this, it is common to observe some body mass increase as a consequence of the drop in female sex hormones, mainly characterized by changes in body composition, with the increased deposition of adipose tissue in different regions [32]. In fact, after 12 weeks of hypoestrogenism, there was an increase in the perigonadal and subcutaneous tissue mass. This corroborates Lino et al. [4], who verified a significant increase of 36.9% in the mesentery and 9.2% (not significant) in the genital region when comparing the group under hypoestrogenism with the control group after 12 weeks. In our study, in the peritoneal region, there was an increase of 43.4% in the OVX group in relation to the CT, although it was not significant. To understand these findings, in the presence of estrogens, there is the activation of ER-α, resulting in a decrease in visceral fat synthesis by decreasing the uptake of fatty acids by the tissue [33]. From this, it is possible to deduce that due to the lower signaling previously mediated by the hormone, the deposition of perigonadal fat was increased. This is concerning because the visceral fat is strongly associated with the onset of diabetes and cardiovascular disease [5,33].

In addition to the relationships with metabolic diseases, excessive fat accumulation can generate inflammatory responses by releasing IL-6 into the circulation [6], while it releases IL-10 as an anti-inflammatory strategy [10]. However, it is not known how much accumulation of adipose tissue needs to occur for such consequences. In fact, this study found that the hypoestrogenism increases IL-6, helping to understand the long-term inflammatory condition generated by an ovarian hormone deficit. In relation to IL-10 levels, although not significant, the OVX group had an increase of 18.9% when compared with CT, possibly linked to the total increase in adipose tissue in this group, seeking to mitigate a possible inflammatory condition. The OVX + MEL group showed a 19.3% increase in IL-10 compared to CT. These findings confirm the relationship about the increase in white adipose tissue present in animals under hypoestrogenism (groups: OVX and OVX + MEL) that raises the serum concentration of this marker, although there is no statistical difference.

In relation to TGF-β1 levels, there was no change after 12 weeks of hypoestrogenism, although an increase in its levels was expected in response to the increase in adipose tissue, seeking to reduce adipogenesis. Possibly the levels of TGF-β1 are sensitive to more significant variations in adipose tissue mass, either an increase or decrease. So, the action of estrogens on TGF-β1 remains unknown, requiring further studies. LTC4 was another variable with limitation in the discussion due to the lack of knowledge in the literature about the influence of hypoestrogenism on circulating levels, although no significant effect was found.

Seeking to avoid excessive fat accumulation, melatonin administration was chosen due to its role in the regulation of lipolysis and suppression of lipogenesis [21]. However, the present study did not find a significant effect of melatonin on the perigonadal, subcutaneous, and peritoneal mass. Meanwhile, the MEL group did not present significant variations in relation to CT on these parameters (29.9%; 10.2%; and 14.2% difference on perigonadal, subcutaneous, and peritoneal, respectively). These findings indicate that despite the chronic experiment model that inevitably conditions confinement and the consequent reduction in activity and fat accumulation, the groups without ovariectomy did not suffer severe alterations. In this context, Hsu et al. [18] administered 10, 20, and 50 mg/kg/day of melatonin via oral gavage to ovariectomized rats, classified as low, medium, and high, respectively. The authors found a reduction in the total mass of the gonadal and perirenal adipose tissue in the animals treated with melatonin, but no dose-dependent effect was found. When evaluating the relativized mass, a reduction in gonadal fat was reported with a high dose of melatonin (50 mg/kg). However, the authors did not include a control group (without ovariectomy), which limits the full understanding that ovariectomy increased adipose tissue mass, although it allows us to affirm that melatonin prevented fat accumulation.

In relation to our study, the potent detrimental effect of hypoestrogenism on visceral and subcutaneous fat was evident, preventing the expected action of melatonin, when identifying an increase of 20.4% and 80.1% in the peritoneal, perigonadal, and subcutaneous region, respectively, of the OVX + MEL group in relation to CT. When comparing the OVX + MEL group with OVX, the fat mass of the peritoneal and perigonadal region was reduced by 15.9% and 6.40%, respectively, although this was not significant. Even so, this small percentage difference is advantageous and allows us to have expectations regarding the role of melatonin in the reduction in adipose tissue in longer studies.

As for inflammatory markers, although there are reports of melatonin’s action in reducing IL-6 levels [8,22] and in increasing IL-10 [20], the present study did not identify significant variations. Melatonin may act in the face of an expressive inflammatory challenge [20], which was not observed within the 12-week period of hypoestrogenism. Still, the OVX + MEL group presented an increase of 19.3% in IL-10 compared to CT, as previously mentioned; however, we cannot say whether it was in response to the greater volume of adipose tissue or by the action of melatonin. Either way, this would be a favorable outcome in an attempt to reduce inflammatory signs.

Regarding TGF-β1, the action of melatonin on its circulating levels is still unknown. In the literature, the reduction in its levels and signaling in cardiac tissue is reported as a strategy to attenuate fibrosis [34] and in the context of chronic obstructive pulmonary disease acting as a protector by reducing TGF-β1 activities [35]. This study would be one of the first to evaluate the influence of melatonin on serum TGF-β1 levels and verify that over 12 weeks, there was no significant effect. In the comparison among the groups, no significant percentage variations were observed (all comparisons below 5%), including in animals under hypoestrogenism. When considering the overall function of TGF-β in having its levels increased to reduce adipogenesis, although this study reported increased adipose tissue in certain regions, there was probably not enough fat accumulation to influence TGF-β1. The understanding of TGF-β is extremely limited in the literature, and further studies are strongly encouraged in the present context.

Regarding the impact of melatonin on anti-inflammatory markers, the literature reveals a significant reduction in the activity of 5-lipoxynegase, which negatively influences the production of leukotriene [23]. On the other hand, our results showed no difference in the LTC4 levels in animals treated with melatonin. Therefore, further studies are still necessary to better understand the indirect and direct impact of melatonin on 5-lipoxynegase and leukotrienes.

In the present study, it was found that melatonin increased the tissue mass of the thymus, an organ with an important function in the immune system, which involutes with advancing age, compromising the production of defense cells and consequently reducing protection against diseases [36]. This result corroborates Bojková et al. [37], who also used exogenous melatonin for 12 weeks and identified its protective function on the tissue, although there was no measurement on the defense cells, as in the present study. 

Specifically in relation to sex steroid hormones and the thymus, the literature has showed an increase in such tissue mass after ovariectomy [38,39,40], but when the procedure is conducted in younger rats than in our study. Possibly the age contributes to our different result, where the thymus was not significantly increased by the estrogen deficit (Figure 4). The literature has also shown that melatonin administration was able to increase thymus weight, but in older mice [41]. Such interesting conditions are not well elucidated in the literature, mainly when we associate the hypoestrogenism with melatonin administration, opening a line of research for future studies.

Despite the methodological care and the maximum scientific basis for discussing the results, this study is not beyond criticism. Firstly, the literature on the topics addressed in this article is still scarce, limiting the consolidated understanding of some findings. In addition, markers related to inflammation were measured exclusively in serum, and, despite relevance when observing the clinical application, it is important to validate them with other tissues. Another relevant observation is regarding the intervention’s duration. Based on the current results, it is suggested that studies lasting more than twelve weeks may generate more significant complications due to the state of hypoestrogenism; while providing a greater challenge, more effects would probably be observed in response to melatonin administration. Also, new studies using different doses of melatonin administration would be valuable. In addition, it would be interesting to use a larger sample size per group, increasing the robustness of the results and consequently strengthening the interpretation, especially in relation to the interactions of statistical effects. In any case, our study is based on the “3 Rs Principle” in animal experimentation (substitution, reduction, and refinement), seeking to minimize the number of animals without compromising the statistical analysis.

## 5. Conclusions

In summary, hypoestrogenism is associated with increased white fat accumulation, mainly in the subcutaneous and perigonadal regions. This increased deposition may potentially be related to the increased release of serum IL-6. Although it is only one pro-inflammatory marker, this result serves as a warning about the possible triggering of an inflammatory condition in the future. In this study, hypoestrogenism did not contribute to a sufficient negative impact on the evaluated parameters for melatonin to provide certain benefits. Although the evaluation of thymus tissue mass is not one of the main targets of this study, it was identified that melatonin increases thymus mass, which potentially improves the immune response to challenges, especially in situations with advanced age. In the future, more extended research is necessary to fully understand the interplay between hypoestrogenism, adipose tissue regulation, and inflammation, as well as melatonin’s potential modulatory role.

## Data Availability

Data are contained within the article.
